# Corinthian Currants Supplementation Restores Serum Polar Phenolic Compounds, Reduces IL-1beta, and Exerts Beneficial Effects on Gut Microbiota in the Streptozotocin-Induced Type-1 Diabetic Rat

**DOI:** 10.3390/metabo13030415

**Published:** 2023-03-11

**Authors:** Vasiliki Kompoura, Ioanna Prapa, Paraskevi B. Vasilakopoulou, Gregoria Mitropoulou, Grigorios Nelios, Evangelos Balafas, Nikolaos Kostomitsopoulos, Antonia Chiou, Vaios T. Karathanos, Eugenia Bezirtzoglou, Yiannis Kourkoutas, Amalia E. Yanni

**Affiliations:** 1 Laboratory of Applied Microbiology and Biotechnology, Department of Molecular Biology and Genetics, Democritus University of Thrace, Dragana, 68100 Alexandroupolis, Greece; 2 Laboratory of Chemistry, Biochemistry, Physical Chemistry of Foods, Department of Nutrition and Dietetics, Harokopio University of Athens, 17671 Athens, Greece; 3 Laboratory Animal Facility, Biomedical Research Foundation of the Academy of Athens, 11527 Athens, Greece; 4 Agricultural Cooperatives’ Union of Aeghion, Corinthou 201, 25100 Aeghion, Greece; 5 Laboratory of Hygiene and Environmental Protection, Department of Medicine, Democritus University of Thrace, Dragana, 68100 Alexandroupolis, Greece

**Keywords:** Corinthian currants, type-1 diabetes, STZ-induced diabetic rat, polar phenolic compounds, inflammation, gut microbiota

## Abstract

The present study aimed at investigating the possible benefits of a dietary intervention with Corinthian currants, a rich source of phenolic compounds, on type 1 diabetes (T1D) using the animal model of the streptozotocin-(STZ)-induced diabetic rat. Male Wistar rats were randomly assigned into four groups: control animals, which received a control diet (CD) or a diet supplemented with 10% *w*/*w* Corinthian currants (CCD), and diabetic animals, which received a control diet (DCD) or a currant diet (DCCD) for 4 weeks. Plasma biochemical parameters, insulin, polar phenolic compounds, and inflammatory factors were determined. Microbiota populations in tissue and intestinal fluid of the caecum, as well as fecal microbiota populations and short-chain fatty acids (SCFAs), were measured. Fecal microbiota was further analyzed by 16S rRNA sequencing. The results of the study showed that a Corinthian currant-supplemented diet restored serum polar phenolic compounds and decreased interleukin-1b (IL-1b) (*p* < 0.05) both in control and diabetic animals. Increased caecal lactobacilli counts (*p* < 0.05) and maintenance of enterococci levels within normal range were observed in the intestinal fluid of the DCCD group (*p* < 0.05 compared to DCD). Higher acetic acid levels were detected in the feces of diabetic rats that received the currant diet compared to the animals that received the control diet (*p* < 0.05). Corinthian currant could serve as a beneficial dietary component in the condition of T1D based on the results coming from the animal model of the STZ-induced T1D rat.

## 1. Introduction

According to the International Diabetes Federation (IDF) [1], in the last 20 years, the number of people suffering from diabetes has almost tripled, making it the most common and prevalent disease in developed countries. While type 2 diabetes (T2D) is the most prevalent, a steep increase in the incidence of type 1 diabetes (T1D) has also been observed. T1D is characterized by an autoimmune-mediated beta-cell destruction of pancreatic islets, resulting in a total deficiency of insulin, while immune and inflammatory cells within and around the islets are present [2]. The exact mechanisms responsible for the pathophysiology of the disease remain unclear; interactions of predisposing genes with the modern, westernized environment are thought to be the main determinants of the onset and development of the disease [3,4].

Studies in animal models and humans have shown a persistent link between gut microbial dysbiosis and a range of metabolic and immunological diseases [5,6], including diabetes [7], inflammatory bowel diseases (IBD) [8,9], irritable bowel syndrome (IBS) [10], obesity [11], cancer [12], cardiovascular [13], and central nervous system disorders [14,15]. Certainly, gut microbiota has been recognized as one of the key environmental factors that contribute to T1D. The involved mechanisms are not fully understood, but they seem to be associated with the high intestinal permeability that is observed in T1D [16]. Changes in the gut microbiota population can contribute to inflammation and altered immune responses, which in turn can affect beta-cell function. Lower microbial diversity, a relative abundance of specific genera of microorganisms, and an altered metabolic profile are characteristics that are linked to the pathogenesis of T1D [16]. It is not yet clear whether inflammation of the intestinal mucosa is a cause or a result of the dysbiotic environment in metabolic disorders such as diabetes [17]. The abundance of certain opportunistic bacteria, i.e., *Escherichia coli*, *Clostridium*, and *Enterococcus* species, is evident and highly associated with inflammation and dysbiosis-driven disorders [18]. Εnhanced growth of certain strains of probiotic bacteria can act as facilitators through lactose fermentation and restore the intestinal pH, antagonizing colonization by pathogenic bacteria at the same time [19]. The most common probiotic strains belong to the *Lactobacillus*, *Bacillus,* and *Bifidobacterium* species, and their presence in the gut improves the host’s overall health by directly inhibiting pathogenic bacteria’s virulence genes or by communicating with the immune system and triggering anti-inflammatory cascades [20].

Diet is a modifiable factor of pivotal importance that influences the gut microbiota ecosystem and immune responses related to T1D [21]. Currants, raisins, and other grape-related products are rich sources of polar phenolic moieties, which have been shown to beneficially affect the condition of diabetes through antioxidant, anti-inflammatory, and immune modulating properties [22,23]. Indeed, several studies have demonstrated the positive effect of currants on health, exhibiting antioxidant [24], antidiabetic [25], cardiovascular protective [26] properties, and gut-microbiota [27] regulating activities on both humans and animals.

Corinthian currants (*Vitis vinifera* L., var. Apyrena) are dried grape fruits that have been grown and harvested for thousands of years in the area of northern Peloponnese, Greece. They are small, deep blue-colored, and sun-dried berries that are an essential part of the Mediterranean diet and have been consumed since antiquity. Research has demonstrated that Corinthian currants possess substantial quantities of polar phenolic compounds; various hydroxy-cinnamic acids, benzoic acids, phenylacetic acids, as well as anthocyanins and other flavonoids, have been identified and quantified in Corinthian currants [28,29,30,31]. They possess a moderate glycemic index and do not affect blood glucose levels, as has been shown by clinical studies conducted in healthy subjects and patients with T2D [32], as well as in studies in animal models [33]. Incorporation of Corinthian currants in a balanced daily dietary pattern provides health benefits associated with cardiovascular disease prevention, such as reduction of blood pressure and cholesterol concentration [32]. Administration of currants to hypercholesterolemic rabbits lowered lipid peroxidation in plasma and inhibited fatty streak initiation in the aorta [33].

Flavonoids and phenolic acids act as preventive agents for multiple metabolic disorders [34]. One possible mechanism to support this function is the neutralization of nitrogen and oxygen species [35]. In addition, numerous in vivo studies have demonstrated the positive impact of isoflavones and anthocyanins derived from blueberries, raspberries, fava beans, and dark grapes on diabetes [36,37,38,39]. Unabsorbed flavonoids eventually reach the colon, where they are hydrolyzed or fermented by the colonic microbiota [40].

Short-chain fatty acids (SCFAs), mainly acetate, propionate, and butyrate, are produced by bacterial fermentation in the gut and exert several effects on the host’s metabolism and immune system. Gut enterocytes absorb the microbiota-produced SCFAs and facilitate their transmission in the blood circulation [41]. Circulating SCFAs influence glucose storage in multiple sites, such as muscle and liver [42]. Of note, acetate administration has been related to the activation of acetyl coenzyme A (acetyl-CoA) carboxylase and alterations in the expression pattern of neurotransmitters that control appetite [43]. SCFAs can interact with the immune regulators of epithelial cells, and their presence can suppress inflammatory pathways [44]. Hence, they are likely to promote the suppression of autoimmunity and T1D [41].

Although there are research studies reporting the beneficial effects of Corinthian currant consumption on the condition of T2D, their effects on T1D have not been examined so far. So, the aim of the present study was to investigate the effects of a 4-week dietary supplementation with Corinthian currants on circulating polar phenolic compounds and inflammatory factors as well as modulation of the gut microbiota in the condition of T1D, using the animal model of the streptozotocin (STZ)-induced diabetic rat.

## 2. Materials and Methods

### 2.1. Animals and Induction of Diabetes

Male Wistar rats (RCCHan: Wistar), initially purchased from Envigo, Italy, and bred in the Laboratory Animal Facility of the Biomedical Research Foundation of the Academy of Athens (BRFAA), participated in the experiments. Fifteen-week-old animals (weighing 380–400 g bw) were intraperitoneally injected with a freshly prepared solution of STZ (Sigma-Aldrich, Hamburg, Germany) in citrate buffer (0.1 M, pH 4.5) in a bolus dose of 60 mg/kg [45,46]. The animals were in a non-fasting state at the time of the injection, which was performed early in the morning. The administered STZ dose was determined according to protocols reported in the literature [45,47,48]. Blood glucose concentration was measured one week after the injection of STZ, ensuring that the condition of diabetes has been established. Glucose measurement was also performed 48 h after the injection in order to prevent fatal hypoglycemia [47,48]. After the first week, animals exhibiting glucose levels above 250 mg/dL [48] accompanied by signs of polyurea and polydipsia were considered diabetic and therefore included in the study. The success rate for the establishment of T1D was 75%. Animal experimentation was reviewed by the Project Evaluation Committee of the BRFAA, approved by the Veterinary Directorate of the Athens Prefecture (Ref. Number 272253/07-04-2021), and carried out in compliance with the European Directive 2010/63.

### 2.2. Dietary Treatment

Twenty-four animals were studied. Rats were individually housed in polysulfone cages of type III IVCs (Blue Line, Tecniplast, Buguggiate, Italy), with 75 air changes per h. Cages were kept in the same animal room with a HEPA-filtered air supply (15 air changes per h) at a controlled room temperature of 21 ± 2 °C, a relative humidity of 55 ± 10%, a 12:12-h light: dark cycle (light period, 07:00 to 19:00), a light intensity of 300 lx at 1 m above the floor, and a positive air pressure of 0.6 Pa within the room. The bedding in each cage was composed of corncobs (Rehofix MK 2000, J. Rettenmaier and Sons, Rosenberg, Germany) and was changed once a week. Before experimentation, animals were individually housed and allowed to acclimate for one week with ad libitum access to filtered tap water and standard rat chow (4RF22, Mucedola, Udine, Italy). All rats in the facility were screened regularly using a health-monitoring program, supervised by the designated veterinarian.

Rats were randomly divided into four groups based on dietary treatments: healthy animals that followed the control diet (n = 6, CD) or the control diet supplemented with 10% *w*/*w* Corinthian currants (n = 6, CCD), and diabetic animals that followed the control diet (n = 6, DCD) or the Corinthian currant diet (n = 6, DCCD). The dietary intervention had a duration of 4 weeks. The duration of the intervention was based on previous studies [45,46,49,50]. The number of animals was calculated according to a power analysis (F-test ANOVA) performed by G*Power 3 software) [51]. The primary outcome was the change in intestinal tissue bifidobacteria (effect size f = 0.711, α = 0.05, power = 0.80). According to this calculation, seven animals per group are needed. However, due to the fact that induction of diabetes was successful in about 75% of the animals, six animals participated in each group. This number was adequate for the detection of significant differences, as it was shown in a previous study [45]. The control diet included standard rat chow, while the Corinthian currant diet was prepared by adding a certain amount of Corinthian currants (kindly provided by the Agricultural Cooperatives’ Union of Aeghion S.A., Aeghion, Greece) to standard rat chow. The amount of Corinthian currants provided to the animals was based on a previous study in animal models [33] and is applicable to the human diet. Taking into consideration the amount of calories provided by currants, 52.6 g (~53 g) of this product (or ~155 kcal) has to be included in a diet of 2000 kcal/day. Diets had similar energy contents. The nutrient composition of the diets is presented in Table 1. Rats received daily a fixed amount of the appropriate dietary treatment (22 g of rat chow for CD and DCD groups; 20 g of rat chow + 2 g of Corinthian currants for CCD and DCCD groups) during the entire experimental period. According to previous studies [28,29], the average content of total phenolics expressed as gallic acid equivalents (GAE) is about 250 mg per 100 g of currants. In addition, in a previous clinical study [32], two servings per day, i.e., 36 g of currants, were provided to subjects with diabetes and showed increased serum antioxidant potential after 24 weeks of intervention. To this end, the amount that should be provided to a person weighing 60 kg, or the human equivalent dose (HED), is about 1.5 mg/kg. The animal equivalent dose (AED), according to Nair and Jacob [52] is calculated to be 6.9 mg/kg, taking into consideration an average rat body weight of 350 g. About 2.5 mg of total phenolics should be received, which is found in ~1 g of currants. It must also be noted that the amount of total phenolics may be lower than 250 mg and that 5 servings of fruits and vegetables are recommended to be consumed per day, so the amount of 2 g of currants/animal per day is reasonable. Every morning, fresh food was provided to the animals according to the above-described diets, and if any chow remained from the previous day, it was removed from the cages.

Full diet composition, including vitamins and minerals, is presented on Table S3. The polar phenolic content of Corinthian currants and the rat chow (Table S1) was determined as previously described [28,53]. Briefly, methanol (MeOH) was used for the extraction of polar phenolics from each food matrix; the solvent was evaporated under vacuo, and the residue was treated with solid phase extraction (SPE). The analyses of the aforesaid samples were performed with ultra-high performance liquid chromatography coupled to a high-resolution mass spectrometer (UHPLC–HRMS) as described in section “UHPLC-HRMS Analysis”.

### 2.3. Sample Collection

Blood samples were collected from the lateral tail vein after 6 h of fasting at the beginning (baseline, week 0) and end (week 4) of the dietary intervention. Heparinized plasma was stored at −80 °C after centrifugation (3000× *g*, 10 min, 4 °C) until analysis. Fresh feces were also collected at the same time points and stored at −20 °C. Every week, animals were weighed and blood glucose was measured by a digital glucose meter (OneTouch Verio FlexTM, Lifescan Ltd., Québec, QC, Canada) in order to ensure that diabetes was established and animals remained in a diabetic state. At the end of the experimental period, animals were euthanized, in random order, by an overdose of isoflurane (ISOVET, Chanelle Pharma, Loughrea, Co. Galway, Ireland). The hindgut intestinal segment (i.e., caecum) of the animals in the four groups was dissected, rinsed with saline, and samples (tissue and intestinal fluid) were taken to be processed for microbiological examination. Caecum was the chosen intestinal segment due to its microbial diversity and abundance [54,55]. Intestinal tissue and fluid samples were diluted 1:1, immediately after being aseptically removed, in 25% glycerol-Ringer’s solution and stored at −80 °C until microbiological analysis.

### 2.4. Blood Analyses

#### 2.4.1. Biochemical Parameters, Insulin, and Inflammatory Factors

Plasma glucose and classic biochemical parameters were determined at the beginning and end of the dietary intervention by using an automated biochemical analyzer (Konelab 60i, Thermo Fisher Scientific Inc., Waltham, MA, USA) and commercially available kits (Thermo Fisher Scientific Inc., Waltham, MA, USA). Plasma insulin was measured by a rat insulin ELISA kit (EZRMI-13K, Merck Millipore, Darmstadt, Germany). Inflammatory markers, i.e., interleukin-1beta (IL-1b) and interleukin-6 (IL-6), were also determined by a sandwich ELISA method (rat IL-1 beta ELISA kit EA100264, and rat IL-6 ELISA kit EA100168, OriGene, Rockville, MD, USA) at the end of the intervention.

#### 2.4.2. Determination of Polar Phenols in Rat Serum

##### Polar Phenol Isolation from Serum Samples

For the extraction of polar phenols, rat serum samples underwent enzymatic hydrolysis prior to liquid-liquid extraction (LLE) with ethyl acetate (EtOAc), as reported by Vasilakopoulou et al. [31]. 100 μL of sodium acetate (CH_3_COONa, 0.1 M, pH 5, Thermo Fisher Scientific, MA, USA ) were added to 100 μL of serum samples. Several 20-μL aliquots of β-glucuronidase/ sulfatase (2000/40 U) were injected into the serum samples; the samples were vortexed and incubated for 45 min in a heating bath that was set at 37 °C. To end the enzymatic hydrolysis, 100 μL of phosphoric acid (H_3_PO_4_, 4% *v*/*v*, Thermo Fisher Scientific, MA, USA) were added to the incubated samples. The samples then underwent further treatment with LLE () with the addition of 700 μL EtOAc (Thermo Fisher Scientific, Pittsburgh, PA, USA) to the serum samples. The samples were then vortexed, centrifuged for 5 min at 5000× *g*, and their supernatants were collected. The extraction process was carried out three times in total, with the supernatants being pooled, evaporated, and reconstituted in 100 μL methanol-water (MeOH-H_2_O, 1:1, *v*/*v*, Thermo Fisher Scientific, Pittsburgh, PA, USA), which were analyzed with UHPLC–HRMS (Thermo Fisher Scientific, Bremen, Germany).

##### UHPLC–HRMS Analysis

As previously reported [53], serum samples were analyzed with UHPLC–HRMS. A binary solvent system consisting of (A) 0.1% formic acid (HCOOH) in acetonitrile (CH_3_CN) and (B) 0.1% *v*/*v* HCOOH was used for chromatographic analysis of polar phenolics, along with an Accucore™ column (C18, 150 × 2.1 mm, 2.6 μm, Thermo Fisher Scientific, Waltham, MA, USA). Gradient elution began with 5% of solvent B for 1.1 min, followed by a rapid increase to 25% in 1.1 min, which then remained isocratic for an additional 1.1 min. After 0.6 min, the concentration of B was raised to 40% and remained at this level for 2.6 min. In the next 3 min, the concentration of B increased to 95%. The initial conditions were then restored in 0.4 min and maintained for 5.6 min for column equilibration, which resulted in a total run time of 18 min; the injection volume was 5 μL. HRMS data were acquired with an Exactive Plus™ Orbitrap mass spectrometer (Thermo Fisher Scientific, Bremen, Germany), which operated in negative heated electrospray ionization mode (H-ESI). Thermo XCalibur 4.0 was used to handle acquisition, while quantitative results were obtained with TraceFinder™ 4.1 (Thermo Fisher Scientific, San Jose, CA, USA).

### 2.5. Analysis of Fecal Microbiota

Feces (700–1200 mg) were homogenized with sterilized buffered peptone water 0.1% (LaB M, Heywood, UK) and were 10-fold diluted using ¼ strength Ringer’s solution (LaB M). The following tests on microbiological analysis were performed: (i) total aerobic counts (TAC) on plate count agar (Laboratorios CONDA, Madrid, Spain) at 30 °C for 72 h, (ii) staphylococci on Baird Parker (Laboratorios CONDA) enriched with egg yolk tellurite (Laboratorios CONDA) at 37 °C for 48 h, (iii) coliforms on Chromogenic Coliform agar (Laboratorios CONDA) at 30 °C for 24 h, (iv) *Enterobacteriaceae* on Violet Red Bile Glucose agar (Lab M) at 37 °C for 24 h, (v) enterococci on Kanamycin Aesculin Azide confirmatory agar (Laboratorios CONDA) at 37 °C for 48 h, (vi) lactobacilli (Gram-positive) on MRS agar (Laboratorios CONDA) at 37 °C for 72 h, (vii) *Clostridium* on Tryptose Sulfite Cycloserine agar (Laboratorios CONDA) enriched with egg yolk tellurite (Laboratorios CONDA) at 37 °C anaerobically for 48 h (Merck Millipore Anaerobic Jar 2.5 L, Oxoid AnaeroGen 2.5 L Sachets), (viii) *E. coli* on Tryptone Bile-X chromogenic agar (LaB M) at 37 °C for 24 h, (ix) bifidobacteria on TOS Propionate agar (Laboratorios CONDA), enriched with MUP supplement (Laboratorios CONDA), at 37 °C for 48 h anaerobically (Merck Millipore Anaerobic Jar 2.5 L, Oxoid AnaeroGen 2.5 L Sachets). All incubations were further extended up to 120 h, but no extra colonies were observed. Results are presented as the log of mean colony-forming units on solid medium culture plates containing between 30 and 300 colonies per gram of fecal samples.

### 2.6. Analysis of Intestinal Tissue Adherent and Intestinal Fluid Microbiota

Intestinal tissue and intestinal fluid of the caecum were homogenized with sterilized buffered peptone water 0.1% (LaB M), vigorously shaken, and subjected to 10-fold dilutions using ¼ strength Ringer’s solution (LaB M). The microbiological tests to evaluate the different microbial populations were performed as described in Section 2.5.

### 2.7. DNA Extraction, PCR Amplification, and 16S rRNA Sequencing

DNA isolation was performed on duplicate fecal samples of each group (CD, CCD, DCD, and DCCD) collected at the baseline and the 4th week. Total DNA was extracted using the NucleoSpin Stool Mini Kit (Macherey and Nagel, Düren, Germany), according to the manufacturer’s instructions, in an elution volume of 100 μL. Next-generation sequencing (NGS) was performed at the Molecular Research DNA laboratory (MR DNA, Shallowater, TX, USA), using the MiSeq platform. PCR primers 27F/519R (AGRGTTTGATCMTGGCTCAG/GTNTTACNGCGGCKGCTG) were used in a 30-cycle PCR using the HotStar Taq Plus Master Mix Kit (Qiagen, Germantown, MD, USA) to target the bacterial 16S rRNA gene for region V1–V4. The following conditions were used to perform the polymerase chain reaction (PCR) amplification: 94 °C for 3 min, 30 cycles of 94 °C for 30 s, 53 °C for 40 s, 72 °C for 1 min, and the final elongation step at 72 °C for 5 min. In order to determine the success of amplification, PCR products were subjected to 2% agarose gel electrophoresis to determine the relative intensity of bands. Then, the amplicons were purified using Ampure XP beads (Beckman Coulter, Brea, CA, USA). An Illumina DNA library using MiSeq sequencing was used for the samples, following the manufacturer’s recommendations. A proprietary analysis pipeline developed by MR DNA was followed for the processing of the sequenced data. Final operational taxonomic units (OTUs) were taxonomically classified using BLASTn against a curated database derived from RDPII and NCBI (www.ncbi.nlm.nih.gov, http://rdp.cme.msu.edu, accessed on 12 December 2022) by clustering at 3% divergence (97% similarity). The analysis of raw data at the OTU level was performed using the Rhea platform [56].

### 2.8. SCFAs

Fecal fatty acid purification, extraction, and determination of SCFA and lactic acid concentrations were performed as previously described [46]. Organic acid concentrations were determined by HPLC using a Shimadzu chromatography system (Shimadzu Corp., Duisburg, Germany) equipped with a Nucleogel ION 300 OA column (Macherey-Nagel), a DGU-20A5R degassing unit, a LC-20AD pump, a CTO-20AC oven at 85 °C, and a RID-10A refractive index detector [57].

Fecal lactic acid and SCFAs concentrations were expressed as mean μmol per g of feces, using the following equation [58]:

(1) $$\mathrm{SCFAs}\;\mathrm{and}\;\mathrm{lactic}\;\mathrm{acid}\;\left(\mu \mathrm{mol}/g\right)=\frac{\mathrm{organic}\;\mathrm{acid}\;\mathrm{in}\;\mathrm{fecal}\;\mathrm{contents}\;\left(\frac{\mathrm{mmol}}{\mathrm{mL}}\right)\times \mathrm{Vd}\;\left(\mathrm{mL}\right)\times 1000}{\mathrm{weight}\;\mathrm{of}\;\mathrm{fecal}\;\mathrm{sample}\;\left(g\right)}\;$$

where Vd is the total volume of dilution of the fecal samples.

### 2.9. Statistical Analysis

Values are expressed as the mean ± SD unless otherwise specified. Statistical analysis was performed using Statistica v. 12 statistical software (StatSoft, Inc., Tulsa, OK, USA). A two-way analysis of variance (ANOVA) coupled with the Bonferroni post-hoc test was used to compare microbiota populations in intestinal tissue and fluid as well as for the inflammatory markers. An ANOVA for repeated measures coupled with the Bonferroni post-hoc test was employed to compare the microbiota populations of fecal samples, blood parameters, and body weight in the four groups of animals. As for the detected levels of polar phenolics, a two-way ANOVA followed by an independent *t*-test was used. Pearson’s correlation coefficient was calculated in order to explore potential correlations between IL-1b and gut microbiota as well as SCFAs. For all statistical analyses, the statistical significance was set at *p* < 0.05.

## 3. Results and Discussion

### 3.1. Effect of Corinthian Currants on Body Weight, Biochemical Parameters, Insulin, and Inflammatory Markers

**Figure 1:**
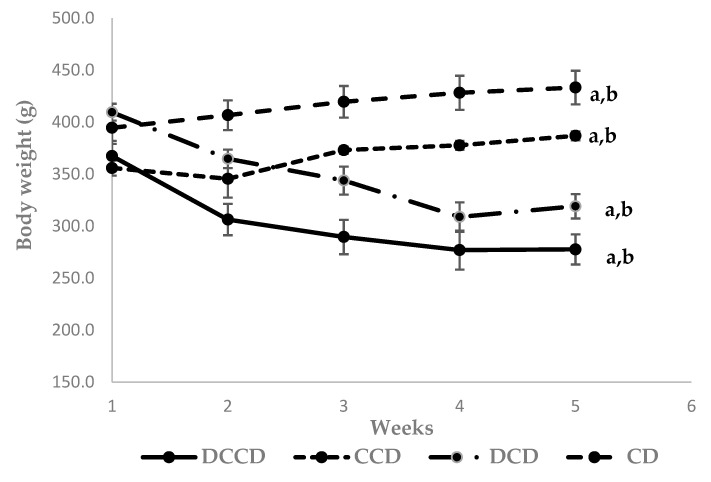
Weekly body weight values (1: baseline-week 0; 5: end-week 4) of control and STZ-induced T1D rats during the dietary intervention. The data are presented as mean ± SEM (n = 6 per group). ^a^ *p* < 0.001 compared to CD, and ^b^ *p* < 0.001 compared to CCD. CD: animals that received the control diet; CCD: animals that received the Corinthian currant diet; DCD: diabetic animals that received the control diet; DCCD: diabetic animals that received the Corinthian currant diet.

After 4 weeks of dietary intervention, the body weight of STZ-induced T1D animals (DCD and DCCD) was significantly decreased compared to the control groups (*p* < 0.001, Figure 1). Diabetic animal models developed polyuria, polyphagia, and polydipsia accompanied by weight loss, symptoms indicative of T1D [34]. The lower *(p* < 0.001) body weight of diabetic animals highlights the catabolic state of poor glycemic control, widely reported in T1D disease and manifested as a result of STZ-induced diabetes [59,60]. Dietary intervention with Corinthian currants did not affect body weight. This is also confirmed by a previous study that evaluated the effect of Corinthian currants on hypercholesterolemia in rabbits. The results demonstrated no significant changes in body weight or animal growth that could be attributed to Corinthian currants administration [33].

At the beginning (week 0) and end (week 4) of the dietary intervention, plasma glucose concentrations were higher in the diabetic groups of animals (DCD and DCCD), compared to the control groups (CD and CCD; *p* < 0.001 in all cases), while they ranged in similar levels between DCD and DCCD, as well as between CD and CCD groups (*p* = 1.000 in all cases). Indeed, glucose levels at baseline were (expressed as mean ± SD, in mg/dL) 112.0 ± 12.8 for CD and 128.0 ± 9.3 for CCD, while they climbed to 332.0 ± 18.3 for DCD and 385.0 ± 23.5 for DCCD after administration of STZ. The concentrations remained stable during the experimental period, and at the end of the 4th week, they were 128.0 ± 17.6, 140.0 ± 24.7, 399.0 ± 48.8, and 407.0 ± 79.0 for CD, CCD, DCD, and DCCD, respectively. Currants contain various nutritional components and possess a low-to-moderate glycemic index, which makes them suitable for consumption by diabetic individuals [32]. A recent study reported lower blood glucose levels and improved glucose tolerance after consumption of blackcurrant extract, but the mechanism underlying this effect remains unclear [61]. Dietary supplementation with Corinthian currants did not affect the plasma lipid profiles of control and diabetic animals.

**Figure 2:**
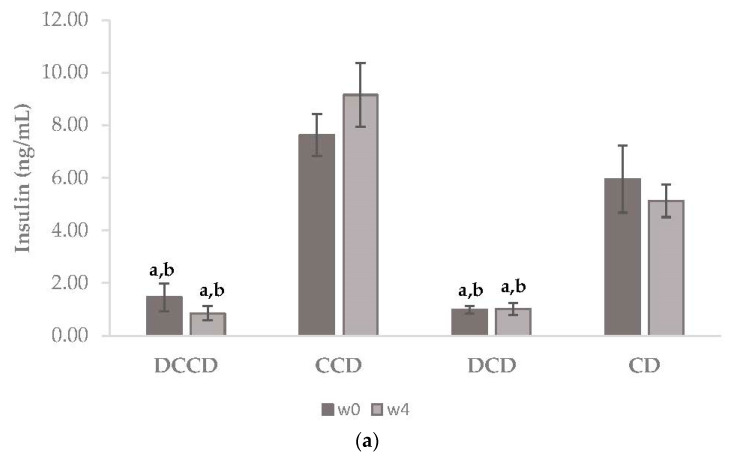

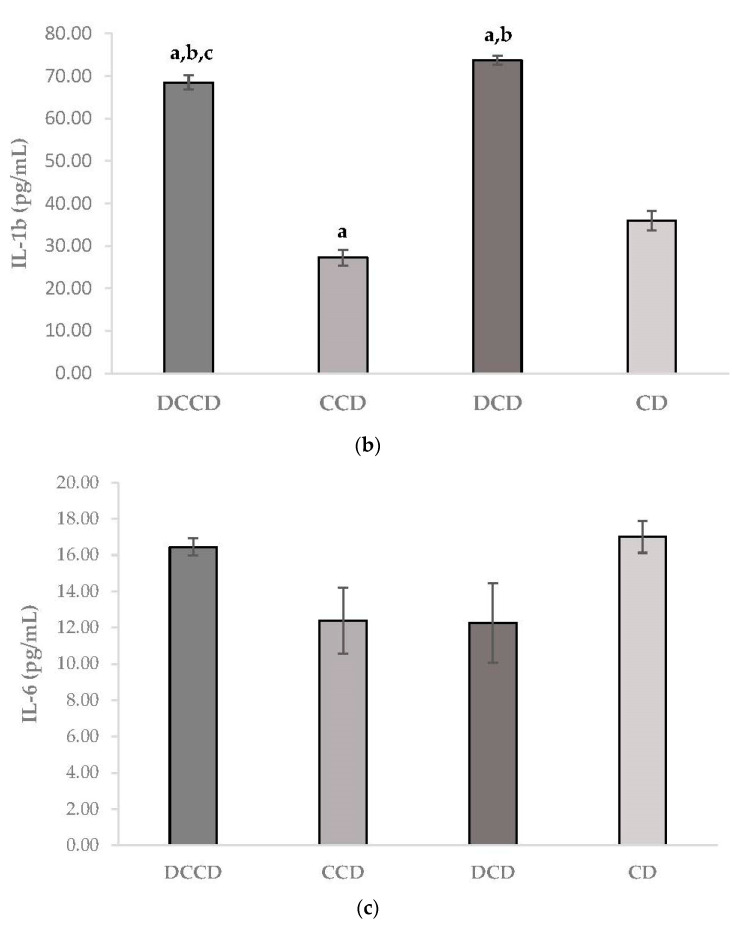
Plasma concentrations of (**a**) insulin, (**b**) IL1-b, and (**c**) IL-6 in control and STZ-induced T1D rats. The data are presented as mean ± SEM (n = 6 per group). ^a^ *p* < 0.01 compared to CD, ^b^ *p* < 0.001 compared to CCD, and ^c^ *p* < 0.05 compared to DCD. CD: animals that received the control diet; CCD: animals that received the Corinthian currant diet; DCD: diabetic animals that received the control diet; DCCD: diabetic animals that received the Corinthian currant diet.

Plasma insulin concentrations were lower in the DCD and DCCD groups (*p* < 0.001), compared to CD and CCD, at the beginning of the study. At the end of the dietary intervention, insulin levels remained low in both diabetic groups (*p* = 0.007 and *p* = 0.001 for DCD and DCCD, respectively). No effects of Corinthian currant consumption were reported regarding insulin levels (Figure 2a). Inflammatory markers were investigated, as they are implicated in the disease of T1D [6,62]. Plasma IL-1b levels were higher in diabetic groups compared to control groups (*p* < 0.001 in all cases, Figure 2b). However, a significant decrease in IL-1b levels was observed in CCD and DCCD compared to CD (*p* = 0.006) and DCD (*p* = 0.048), respectively. Dietary intervention with Corinthian currants managed to lower the values of IL-1b in plasma after 4 weeks, conferring a positive effect on the inflammatory profile. On the other hand, IL-6 remained at similar levels in all groups, and no significant changes were noticed after the dietary intervention with Corinthian currants (*p* = 0.228 between DCD and CD, *p* = 0.433 between DCCD and CCD, *p* = 0.387 between DCCD and DCD, and *p* = 0.257 between CD and CCD, Figure 2c).

In accordance with our results, administration of *Vitis vinifera* products (seed, extract, and fruit) has been shown to decrease inflammatory and apoptotic markers and could possibly account for hepatoprotective properties in diabetes [63]. Furthermore, daily consumption of blackcurrants for longer periods of time may lead to the promotion of antioxidant and anti-inflammatory intracellular events that affect not only metabolic disorders but also overall health [24]. Chang et al. [64] investigated the effect of resveratrol, a phenolic compound found in Corinthian currants, on oxidative stress and the inflammatory response in STZ-induced T1D rats. The results indicated a significant decrease in oxidative stress as well as reduced hepatic inflammation (NF-kB and IL-1b), while no positive effect was recorded on pro-inflammatory markers (TNF-α and IL-6).

Unperformed analyses of inflammatory tissue protein markers and oxidative stress markers present a limitation in the present study. Such analyses must be taken into consideration in future studies, since describing signaling pathways could shed light on the exact mechanisms of the anti-inflammatory and antioxidant effects of Corinthian currants. To our knowledge, these effects have not previously been described in the context of T1D. However, it has been shown that supplementation with polyphenol-rich blackcurrant extract in obese mice resulted in decreased obesity-induced inflammation in adipose tissue and splenocytes, partially through modulation of energy metabolism in muscle tissue. Indeed, when the splenocytes of mice fed with blackcurrant extract were stimulated by lipopolysaccharides, IL-1b mRNA was significantly lower than that of control splenocytes [65]. It has also been reported that blackcurrant anthocyanins suppress the expression and secretion of proinflammatory mediators in macrophages by inhibiting nuclear translocation of NF-kB [66].

### 3.2. Polar Phenolics Detected in Rat Serum

Since a significant amount of polar phenolics found in the bloodstream is in the form of glucuronidated and sulfated conjugates, serum samples of rats were treated with enzymatic hydrolysis [67,68]. On this occasion, the enzymes break the O-glucuronide or O-sulfate bonds [69], liberating the polar phenolic precursors, often referred to as aglycones or free-form polar phenolics, which may then be easily quantified by using various analytical techniques, such as UPLC–HRMS. Given that this methodology appears to provide meaningful information, it is essential to note that this type of information is indirect and only provides a quantitative estimate of polar phenol aglycone levels in vivo [69,70].

A two-way ANOVA showed a statistically significant (*p* < 0.05) interaction among the effects of Corinthian currant administration and STZ treatment for chrysin (*p* < 0.001), isorhamnetin (*p* < 0.001), kaempferol (*p* = 0.016), quercetin (*p* < 0.001), daidzein (*p* < 0.001), vanillic acid (*p* < 0.001), and syringic acid (*p* < 0.001). In addition, significant simple main effects for Corinthian currant intake were found for all detected polar phenolics, besides syringic acid (*p* = 0.083). However, with the exception of hesperetin (*p* = 0.064), naringenin (*p* = 0.370), genistein (*p* = 0.055), and trans-cinnamic acid (*p* = 0.199), the remaining detected polar phenolics demonstrated statistically significant simple main effects for STZ treatment (Table S2). Independent Samples T-test analysis revealed that the circulating levels of all detected flavonoids, along with vanillic and syringic acid, were statistically significantly higher (*p* < 0.05) in the control group that consumed a Corinthian currant-supplemented diet (CCD) compared to the control group that consumed only the typical diet (CD) (Table 2). However, the following were found to be significantly higher in the diabetic group consuming a Corinthian currant-supplemented diet (DCCD) compared to the diabetic group consuming only the typical diet (DCD): apigenin (*p* < 0.001), daidzein (*p* < 0.001), formononetin (*p* < 0.001), genistein (*p* < 0.001), hesperetin (*p* < 0.001), kaempferol (*p* < 0.001), luteolin (*p* < 0.001), naringenin (*p* < 0.001), quercetin (*p* < 0.001), syringic acid (*p* = 0.006), and vanillic acid (*p* < 0.001). 

**Figure 3:**
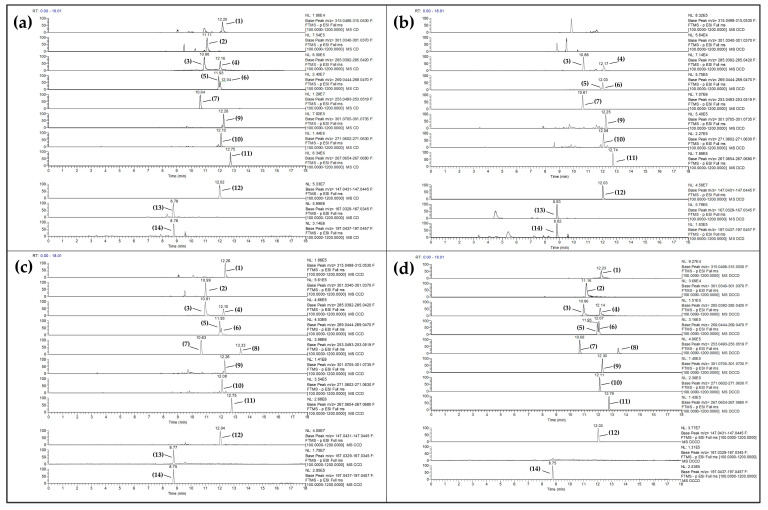
Extracted ion chromatograms (XICs) of polar phenols detected in rat serum; the numbered peaks correspond to: (1) isorhamnetin, (2) quercetin, (3) kaempferol, (4) luteolin, (5) apigenin, (6) genistein, (7) daidzein, (8) chrysin, (9) hesperetin, (10) naringenin, (11) formononetin, (12) trans-cinnamic acid, (13) vanillic acid, and (14) syringic acid. The lowercase letters represent the four distinct groups: (**a**) control group (CD), (**b**) diabetic group (DCD), (**c**) Corinthian currant-supplemented group (CCD), and (**d**) diabetic Corinthian currant-supplemented group (DCCD) (n = 6 per group).

These findings may give insight into the circulating levels of polar phenols, implying that the observed increase in some of them could be the outcome of Corinthian currant consumption on a daily basis over a sustained period of time (Table 2, Figure 3). Flavanones and isoflavones are found to have the best bioavailability profiles among flavonoids [67,71,72,73]. Several pharmacokinetic studies on healthy male rats have shown that the half-lives (T_1/2_) of the aglycones daidzein and genistein are 5–9 h [67,71,72]. As can be seen in Table S1, the rat chow appears to have greater amounts of isoflavones (daidzein, formononetin, and genistein) and some flavones (apigenin and luteolin) than Corinthian currant; however, flavanones (hesperetin and naringenin) were found to be present at similar levels. This could be considered a shortcoming of our study, given that the rats’ chow is plant-based, disallowing a polar phenol-free diet. As a result, the rat chow may hinder the bioavailability of the polar phenols of Corinthian currant in the diet under examination. Understanding bioavailability is particularly challenging due to the fact that natural foods are complex blends of numerous free and conjugated polar phenolics, each having its own bioavailability, metabolism, and excretion profiles. Meanwhile, the most common microbiota catabolites generated as a result of flavonoids and anthocyanins consumption are small-molecule polar phenolic compounds, including many benzoic, phenylacetic, and propionic acids [74]. There is also evidence that the isoflavone aglycones daidzein and genistein can be generated by the metabolic alteration of other existing isoflavone glycosides in the administered diet, by enzymes or gut microflora, prior to being absorbed into circulation [71].

In addition to the above, our results (Table 2, Figure 3) revealed that the detected levels of some flavonoids, namely apigenin, chrysin, isorhamnetin, and quercetin, were considerably higher in the CCD group when compared to the DCCD group (*p* < 0.05); for daidzein, our findings indicate the opposite. However, for formononetin (*p* = 0.05), genistein (*p* = 0.957), hesperetin (*p* = 0.162), kaempferol (*p* = 0.669), luteolin (*p* = 0.056), and naringenin (*p* = 0.861), no significant differences among the CCD and DCCD groups were observed. In addition, most flavonoids were statistically significantly higher (*p* < 0.05) in the CD group than the DCD group.

Polar phenols have various health benefits due to their structural similarities and ability to influence several cellular processes that are relevant to many diseases, including diabetes. These effects are caused by a combination of factors, such as reducing oxidative stress, suppressing inflammation, regulating insulin signaling, glucose metabolism, and lipid levels, among others [75]. These mechanisms may be responsible for the polar phenols’ ability to protect beta cells, increase insulin sensitivity, and lower blood sugar levels. However, it is important to note that in disease states, changes in transporters and enzymes responsible for drug metabolism and gut transit can occur [76,77,78]. Furthermore, studies have shown that the gut microbiota is altered in rats with diabetes induced by a high-fat diet and low-dose STZ, which may reduce the biotransformation of polar phenols due to imbalances in gut bacteria and enzymes [79,80].

Additionally, polar phenols have been found to have anti-diabetic effects by acting as antioxidants through various mechanisms, such as regulating the expression of anti-apoptotic factors, blocking oxidant enzymes, scavenging reactive oxygen species, chelating metal ions that create free radicals, modifying signal transduction pathways and mitochondrial dynamics, and increasing autophagy [75,81]. The free radical scavenging activity of polar phenols has been analyzed both thermodynamically and kinetically. For example, a study by Di Meo et al. [82] explored the structure-activity relationship of quercetin’s free radical scavenging activity from a kinetic perspective by examining three possible mechanisms for H-atom transfer that are likely to occur in living organisms. This sacrificial activity of polar phenolics interrupts free radical chain reactions. This could explain the decreased levels of certain polar phenolics, namely apigenin, chrysin, isorhamnetin, and quercetin, after STZ treatment, which induces diabetes in rats and is therefore under an oxidative stress state; the polar phenolics may be sacrificed in an attempt to attenuate the redox imbalance. The most commonly reported mechanism for the antioxidant properties of polar phenols is their ability to scavenge radicals. They can interrupt free radical chain reactions by acting as sacrificial agents [83]. This sacrificial behavior may play a role in mitigating the redox imbalance seen in diabetes. However, more research is necessary to fully understand how polar phenols’ sacrificial mechanisms may affect oxidative stress in type 1 diabetes and to explain the variations in the levels of polar phenols found in rat serum.

In a nutshell, polar phenolics were detected in rat serum from all groups (Figure 3), reflecting their fasting state in circulating blood. Using enzymatic hydrolysis, the levels of polar phenol aglycones in vivo were estimated, as most of these phytochemicals are present as conjugated derivatives of phase II metabolism. These findings give insight into the levels of polar phenols in the bloodstream and suggest that the observed increase in some polar phenols may be a result of the long-term supplementation with Corinthian currant. On the other hand, the decrease or lack of difference in some polar phenols in diabetic rats may be a result of metabolic imbalances caused by their pathological state.

### 3.3. Analysis of the Fecal Microbiota

Microbiological analysis of fecal samples revealed that TAC did not differ between groups (*p* = 1.000 in all cases) (Table 3). Likewise, staphylococcal, lactobacilli, and *Bifidobacterial* counts ranged at similar levels in all groups with no difference between them (*p* = 0.077 between DCD and CD in staphylococci, *p* = 1.000 in all other cases). Coliforms, *Enterobacteriaceae*, *E. coli,* and *Clostridium* levels were significantly increased in all diabetic groups compared to control groups at baseline (*p* < 0.001, week 0), as well as at week 4 (*p* < 0.001). A significant increase was also observed in enterococcal counts at the 4th week in the DCD group compared to the CD group (*p* = 0.001), while no significant difference was recorded in both groups that received the Corinthian currant diet (CCD and DCCD). Corinthian currant appeared to maintain enterococci levels within a normal range. Being both a commensal and an opportunistic pathogenic bacterial species, maintaining their balance could be of great importance [84]. Thus, the unaffected enterococci levels of diabetic animals after the dietary intervention with Corinthian currants can be perceived as a possible beneficial outcome.

Intestinal inflammation caused by the diabetic condition may lead to an imbalanced microbiota and up-regulated abundances of pathogenic bacteria species, such as *Enterobacteriaceae*, coliforms, and *Bacteroidetes* [85]. A significant increase in *Bacteroidetes* and *Clostridiaceae* abundance has been reported in fecal samples of STZ-induced diabetic Sprague-Dawley rats, while increased levels of bacteria that belong to the genus *Clostridium* in animal feces were also found [86]. Such an alteration in the intestinal microbiota composition was observed after the injection with STZ and could be possibly linked with the manifestation and progression of T1D, as their levels remained elevated even after 5 weeks of experimentation. These results coincide with the findings of our study, which portray the gut dysbiosis derived from T1D.

### 3.4. Analysis of the Intestinal Tissue and Fluid Microbiota

Although in the caecum (intestinal tissue and fluid), similar results as in fecal samples were observed in TAC, coliform, *E. coli*, *Enterobacteriaceae,* and staphylococcal counts, a significant decrease was noticed in enterococcal and clostridial counts in DCCD compared to the DCD group (*p* < 0.001, Table 4), while elevated enterococci levels were reported in DCD compared to CD and CCD (*p* < 0.001 in both cases). Corinthian currant supplementation for four weeks resulted in increased lactobacilli counts in both the CCD and DCCD groups compared to the CD and DCD groups (*p* < 0.001) in caecum intestinal fluid samples. Marteau et al. [87] studied the differences between fecal and caecal microbiota. Their findings suggest that the human caecal microbiota differs quantitatively and qualitatively from the fecal microbiota. It harbors fewer anaerobes, while facultative anaerobes represent an important part, and it would therefore be more appropriate to study the colonic microbiota rather than feces for functions occurring in the caecum, such as the fermentation of dietary fibers and endogenous substrates.

Bacteria belonging to the genera *Lactobacillus* and *Bifidobacterium* are studied for their beneficial health effects in various conditions and metabolic diseases, such as diabetes, obesity, and hypercholesterolemia, as well as in maintaining a general health status [88,89]. In a simulated human digestion system study, sun-dried currant digestion resulted in increased lactobacilli and bifidobacterial counts [90], which is in accordance with our results as well as those presented by another study regarding diet supplementation with currants in animal models [91].

Lactobacilli comprise a large number of lactic acid bacteria and have been associated with a wide range of health-promoting effects, including antimicrobial, antioxidant, anti-allergenic, and anti-diabetic, among others [92]. The increased lactobacilli levels could potentially have a regulatory role in the progression of T1D.

### 3.5. Fecal Microbiota Determined by Next-Generation DNA Sequencing

In the feces of both diabetic and control rats, Firmicutes and Bacteroidetes were determined as the dominant phyla with relative abundances of >70% and >19%, respectively (Table 5). Overall, the relative abundances of Firmicutes, Bacteroidetes, Actinobacteria, and Verrucomicrobia were affected neither by the presence of T1D nor by the Corinthian currant supplementation. On the contrary, a significant increase (*p* = 0.007) in the relative abundance of Tenericutes was recorded in the CCD group after the 4-week Corinthian currant supplementation, compared to baseline levels. However, Corinthian currant did not result in increased levels of Tenericutes in the feces of the diabetic group DCCD, which remained similar to baseline levels (*p* = 1.000 in all cases).

The phylum Tenericutes, recently reclassified into a Bacilli clade of Firmicutes [93], is composed of bacteria characterized by the absence of a peptidoglycan cell wall [94]. The majority of Tenericutes have been reported as either commensal or obligate parasites of domestic animals, plants, insects, and humans [95]. According to a recent metagenomic analysis, over 100 uncultured Tenericutes were discovered in the human gastrointestinal tract [96], but it still remains unclear whether these novel lineages are contributing to the maintenance of gut homeostasis and the overall microbiome function [97]. Regarding the presence of Tenericutes in T1D, lower relative abundances have been previously reported in humans with T1D and in non-obese T1D mice compared to non-diabetic controls [98,99]. Additionally, lower abundances of Tenericutes have been observed in diet-induced obese mice [100], in T2D mice after inulin administration [101], and in T2D patients with cognitive impairment [102]. According to the results of the present study, Tenericutes levels in the feces of T1D rats did not seem to be affected by the disease and were increased after Corinthian currant supplementation only in control animals.

**Figure 4:**
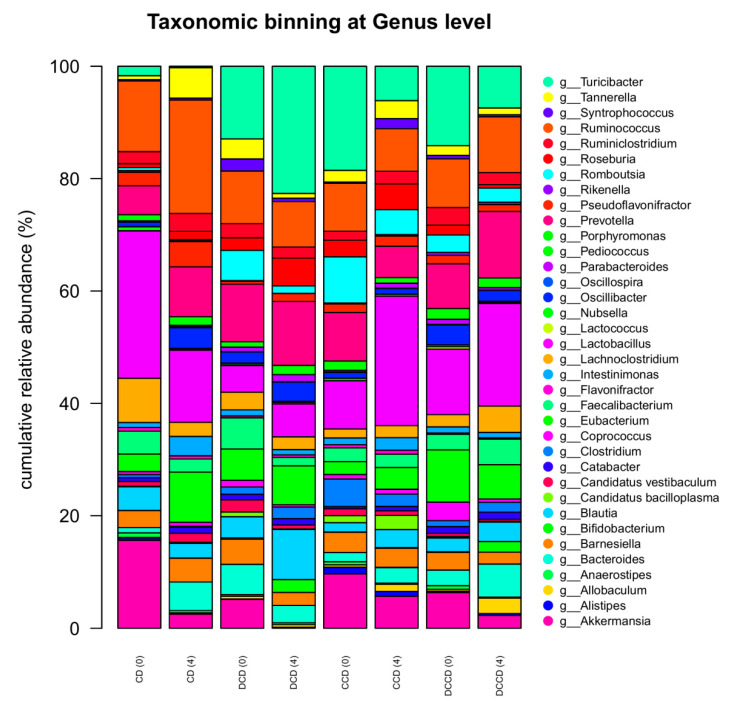
Taxonomic-binning at the genus level with normalized relative abundances (%) after four weeks of Corinthian currant supplementation in control and STZ-induced diabetic rat feces, as determined after 16S rRNA NGS (n = 2 per group). CD: animals that followed the control diet; CCD: animals that followed the Corinthian currant diet; DCD: diabetic animals that followed the control diet; DCCD: diabetic animals that followed the Corinthian currant diet. Parenthesis indicating the timepoint of the diet, 0: baseline week, and 4: after 4 weeks of dietary intervention with Corinthian currant.

At the genus level (Figure 4), a significant increase (*p* = 0.031) in *Candidatus bacilloplasma* levels was observed in the feces of the CCD group. *Candidatus bacilloplasma* belongs taxonomically to the Tenericutes phylum, and the observed increase in its levels was in accordance with the correspondingly increased levels of Tenericutes. Notably, *Candidatus bacilloplasma* has been reported as an opportunistic pathogen associated with gastrointestinal diseases in aquatic organisms [97,103], but such findings have not been confirmed in either humans or rodents. In addition, increased relative abundances of *Eubacterium* and *Coprococcus* were recorded in the feces of diabetic rats compared to the control group, but only in the case of *Coprococcus* was this increase statistically significant (*p* = 0.026). This result is in contrast with human studies, which reported a trend toward a reduction in the levels of *Coprococcus* in T1D individuals [104]. In general, the bacteria of the *Coprococcus* genus have been linked with many beneficial effects for the host, such as the reinforcement of the intestinal epithelial barrier function and intestinal immunity, nutrient supplementation, and the production of anti-inflammatory SCFAs [103,105,106,107,108]. However, the 4-week Corinthian currant supplementation did not affect the relative abundances of this genus in both control and diabetic groups (*p* = 1.000 in all cases).

### 3.6. SCFAs

According to our results, dietary intervention with Corinthian currants altered the levels of SCFAs as presented in Table 6. Increased levels of acetic acid were detected in the feces of the CCD and DCCD groups in the 4th week of the study compared to their respective control groups and baseline levels. However, only in the case of the DCCD group was this result significant (*p* < 0.001 vs. DCD, *p* = 0.011 vs. baseline). Additionally, increased levels of isobutyric acid were observed in the 4th week of the study in all groups compared to baseline levels (*p* < 0.001 in CD, *p* < 0.001 in CCD, *p* = 0.027 in DCD, and *p* < 0.001 in DCCD). Regarding the levels of propionic, butyric, isovaleric, valeric, and lactic acids, no significant difference among the four groups was observed.

The term SCFAs refers to volatile carboxylic acids with 1–6 carbon atoms that are the products of gut microbial fermentation of dietary fiber carbohydrates in the large intestine [109,110,111]. The list of SCFAs includes formic, acetic, propionic, butyric, isobutyric, valeric, isovaleric, and hexanoic acids, with acetic, propionic, and butyric acids being the most prevalent in the intestine at levels of 90 to 95% [112]. The majority of SCFAs produced are absorbed and utilized in the intestinal tract, and only 5–10% are estimated to be excreted in feces [111].

Extensive research on SCFAs has demonstrated their significant role in intestinal homeostasis and the development of metabolic diseases such as T1D and T2D. Acetic acid, predominantly produced by bacteria belonging to the phylum Bacteroidetes [113], has been strongly associated with the regulation of insulin sensitivity and body weight by affecting glucose homeostasis and lipid metabolism [114]. Notably, in our study, Corinthian currant supplementation led to increased levels of acetic acid in the feces of diabetic rats (56.08 μmol/g) compared to their respective control group (24.49 μmol/g) and baseline levels (34.79 μmol/g). A possible explanation for this result might lie in the presence of fructooligosaccharides (FOS) in currants [90], which, as has been reported, can promote the production of acetic acid by the intestinal flora [115,116,117]. However, supplementation with Corinthian currants did not seem to alter the levels of lactic acid and the rest of the SCFAs determined, including isobutyric acid, whose levels were increased at the end of the 4-week study period in all groups.

### 3.7. Potential Correlations between Circulating Inflammatory Markers, Gut Microbiota, and SCFAs

A negative correlation between plasma IL-1b and lactobacilli counts in the intestinal fluid of the DCCD group was detected (*p* = 0.003), while no correlation was observed between plasma IL-1b and SCFAs. However, measurements of intestinal tissue inflammatory biomarkers could give safer results regarding correlations with gut microbiota and SCFAs compared to serum biomarkers. Hence, further studies are needed in order to support any association.

## 4. Conclusions

Type 1 diabetes (T1D) is becoming a major public health concern worldwide as its prevalence has been increasing rapidly. Beyond predisposing genes, environmental components contribute to the onset and development of the disease. Diet is a modifiable factor of pivotal importance that can affect parameters linked with T1D such as oxidative stress, inflammation, and the quality of gut microbiota. Corinthian currants (*Vitis vinifera* L., var. Apyrena) are dried grape fruits, which constitute an integral part of the Mediterranean diet and have been consumed since antiquity in the area of northern Peloponnese, Greece. They are a rich source of phenolic compounds, and several studies have demonstrated their positive health effects, including antioxidant, anti-inflammatory, and cardioprotective properties, as well as beneficial effects on patients with T2D. The present study aimed to investigate the possible impact of Corinthian currant consumption on T1D. For this reason, the STZ-induced T1D rat was used as animal model. The results of the present study showed that a Corinthian currant-supplemented diet restored polar phenolic compounds in serum and decreased the inflammatory factor IL-1b in diabetic animals. Our findings suggest that the daily consumption of a Corinthian currant-supplemented diet over a sustained period of time led to heightened levels of some circulating polar phenolics; however, the reduction or absence of change in certain polar phenols in diabetic rats may be due to the metabolic disturbances caused by their disease condition. It would appear that Corinthian currant acted directly through its constituents’ properties and indirectly through minor alterations in the gut microbiota composition. Furthermore, the dietary intervention had a regulatory effect on enterococci levels and a beneficial effect on caecal lactobacilli counts. The levels of acetic acid, which has been associated with insulin sensitivity, glucose, and lipid homeostasis, were increased in the feces of diabetic animals that followed the currant diet. Although minor, the changes in metabolism and microbiota diversity suggest that Corinthian currant could serve as a beneficial dietary approach in the condition of T1D based on the results coming from the animal model of the STZ-induced T1D rat. Further investigation is needed in order to determine the duration and dose of currant administration, as well as carefully designed clinical studies that would take into consideration the differences in gut microbiota between humans and rodents. Yet, it is not fully understood how different dietary approaches affect the intestinal environment, and future studies might provide a useful insight.

## Figures and Tables

**Table 1:** Nutrient composition and energy content of diets provided to the animals (g per 100 g).

	Control Diet	Corinthian Currant Diet
Proteins (g)	18.5	17.0
Fats (g)	3.0	2.7
Carbohydrates (g)	46.3	49.0
Dietary fiber (g)	6.0	6.1
Currants (g)	-	10.0
Energy (Kcal)	387.6	380.8

Corinthian currants contain 2.9 g of proteins, 73 g of carbohydrates, and 6.8 g of dietary fiber per 100 g.

**Table 2:** Polar phenols quantified in rat serum samples.

Polar Phenol (ng/mL Serum)	CD	DCD	CCD	DCCD
**Flavonols**				
Isorhamnetin	tr	n.d.	6.45 ± 1.65 ^a^	tr ^b^
Kaempferol	3.37 ± 2.35	tr ^a^	8.13 ± 1.6 ^a^	7.86 ± 0 ^c^
Quercetin	0.57 ± 0.13	n.d. ^a^	9.58 ± 3.75 ^a^	0.66 ± 0.05 ^b,c^
**Flavones**				
Apigenin	40.96 ± 10.63	0.93 ± 0.44 ^a^	189.75 ± 40.87 ^a^	130.8 ± 14.55 ^b,c^
Chrysin	n.d.	n.d.	2.18 ± 0.51 ^a^	tr ^b^
Luteolin	5.56 ± 2.03	1.02 ± 0.7 ^a^	125.69 ± 54.07 ^a^	77.51 ± 26.82 ^c^
**Flavanones**				
Hesperetin	2.13 ± 0.45	1.36 ± 1.32	10.82 ± 2.63 ^a^	8.97 ± 1.97 ^c^
Naringenin	3.45 ± 0.48	1.78 ± 1.22 ^a^	9.33 ± 2.18 ^a^	9.59 ± 3.19 ^c^
**Isoflavones**				
Daidzein	5.07 ± 1.32	3.3 ± 1.81	18.12 ± 5.35 ^a^	27.51 ± 6.56 ^b,c^
Formononetin	30.93 ± 3.43	2.14 ± 1.85 ^a^	93.35 ± 22.32 ^a^	66.85 ± 23.27 ^c^
Genistein	14.91 ± 1.69	1.25 ± 0.93 ^a^	42.74 ± 13.91 ^a^	42.35 ± 11.92 ^c^
**Benzoic acid derivatives**				
Vanillic acid	684.37 ± 12.49	807.2 ± 88.93 ^a^	937.59 ± 131.57 ^a^	n.d. ^b^
Syringic acid	13.53 ± 1.18	24.78 ± 13.1	45.12 ± 8.92 ^a^	4.08 ± 0.9 ^b,c^
**Cinnamic acid derivatives**				
Trans-Cinnamic acid	190.02 ± 81.23	167.45 ± 83.02	278.22 ± 93.87	397 ± 122 ^c^

Serum samples were compared with independent sample *t*-tests. Data are expressed as mean ± SD (n = 6 per group), ^a^ *p* < 0.05 compared to CD, ^b^ *p* < 0.05 compared to CCD, and ^c^ *p* < 0.05 compared to DCD. Values lower than the limit of quantitation (<LOQ) are stated as traces (tr). Not detected (n.d.). CD: animals that followed the control diet; CCD: animals that followed the Corinthian currant diet; DCD: diabetic animals that followed the control diet; DCCD: diabetic animals that followed the Corinthian currant diet.

**Table 3:** Effect of diets on the fecal microbiota population in control and diabetic animals at baseline and the 4th week of Corinthian currant supplementation.

	CD		CCD		DCD		DCCD	
	Baseline	4w	Baseline	4w	Baseline	4w	Baseline	4w
TAC	8.28 ± 0.41	8.44 ± 0.26	8.38 ± 0.25	8.59 ± 0.36	8.26 ± 0.14	8.49 ± 0.41	8.58 ± 0.21	8.59 ± 0.19
Enterococci	5.98 ± 0.31	6.03 ± 0.32	6.12 ± 0.36	6.04 ± 0.20	6.31 ± 0.23	6.98 ± 0.33 ^a,b^	6.50 ± 0.44	6.61 ± 0.41
Coliforms	4.39 ± 0.52	4.49 ± 0.34	4.85 ± 0.41	4.93 ± 0.31	5.60 ± 0.65 ^a,b^	6.80 ± 0.51 ^a,b^	5.88 ± 0.63 ^a,b^	7.06 ± 0.43 ^a,b^
*Enterobacteriaceae*	4.42 ± 0.55	4.59 ± 0.35	4.90 ± 0.41	4.94 ± 0.31	5.69 ± 0.62 ^a,b^	6.85 ± 0.47 ^a,b^	5.87 ± 0.60 ^a,b^	7.04 ± 0.44 ^a,b^
Staphylococci	6.28 ± 0.28	5.95 ± 0.29	6.68 ± 0.28	6.29 ± 0.26	6.11 ± 0.20	7.07 ± 0.32	6.48 ± 0.67	6.52 ± 0.62
*E. coli*	4.33 ± 0.65	4.32 ± 0.43	4.77 ± 0.44	4.78 ± 0.29	5.41 ± 0.73 ^a,b^	6.48 ± 0.53 ^a,b^	5.86 ± 0.63 ^a,b^	6.78 ± 0.33 ^a,b^
Clostridia	6.91 ± 0.31	6.89 ± 0.54	6.75 ± 0.3	6.96 ± 0.34	7.82 ± 0.27 ^a,b^	8.27 ± 0.44 ^a,b^	8.48 ± 0.15 ^a,b^	8.06 ± 0.20 ^a,b^
Bifidobacteria	8.25 ± 0.14	8.25 ± 0.36	8.28 ± 0.26	8.30 ± 0.32	8.25 ± 0.26	8.46 ± 0.36	8.29 ± 0.29	8.79 ± 0.32
Lactobacilli	8.47 ± 0.36	8.69 ± 0.22	8.65 ± 0.12	8.72 ± 0.26	8.36 ± 0.05	8.48 ± 0.36	8.54 ± 0.28	8.56 ± 0.26

Data are expressed as mean ± SD (n = 6 per group). ^a^ *p* < 0.01 compared to CD; ^b^ *p* < 0.001 compared to CCD. CD: animals that followed the control diet; CCD: animals that followed the Corinthian currant diet; DCD: diabetic animals that followed the control diet; DCCD: diabetic animals that followed the Corinthian currant diet; TAC: total aerobic counts.

**Table 4:** Effect of diets on the gut microbiota populations of control and diabetic animals in intestinal fluid and intestinal tissue of the caecum.

	CD		CCD		DCD		DCCD	
	Intestinal Fluid	Intestinal Tissue (Caecum)	Intestinal Fluid	Intestinal Tissue (Caecum)	Intestinal Fluid	Intestinal Tissue (Caecum)	Intestinal Fluid	Intestinal Tissue (Caecum)
TAC	6.21 ± 0.10	5.63 ± 0.38	5.26 ± 0.05	6.11 ± 0.07	6.42 ± 0.06 ^b^	6.42 ± 0.46	6.81 ± 0.31 ^a,b^	5.93 ± 0.55
Enterococci	4.71 ± 0.21	3.53 ± 0.49	4.48 ± 0.38	3.52 ± 0.24	6.25 ± 0.19 ^a,b^	3.98 ± 0.84	5.13 ± 0.44 ^c^	4.28 ± 0.48
Coliforms	3.54 ± 0.43	2.18 ± 0.21	3.72 ± 0.15	2.72 ± 0.36	5.20 ± 0.13 ^a,b^	4.32 ± 0.68	5.23 ± 0.36 ^a,b^	3.75 ± 0.89
*Enterobacteriaceae*	3.35 ± 0.31	2.21 ± 0.24	3.79 ± 0.24	2.63 ± 0.34	5.38 ± 0.20 ^a,b^	5.01 ± 0.70	5.77 ± 0.30 ^a,b^	4.80 ± 0.34
Staphylococci	3.58 ± 0.23	3.11 ± 0.24	4.75 ± 0.14	3.13 ± 0.17	5.46 ± 0.10 ^a,b^	4.45 ± 0.07	5.20 ± 0.31 ^a,^	3.95 ± 0.95
*E. coli*	3.34 ± 0.11	2.15 ± 0.25	3.74 ± 0.16	2.49 ± 0.18	5.34 ± 0.19 ^a,b^	4.55 ± 0.80	4.65 ± 0.72 ^a^	3.69 ± 1.10
Clostridia	5.94 ± 0.20	4.10 ± 0.44	6.00 ± 0.16	4.96 ± 0.31	7.36 ± 0.10	5.35 ± 0.40	5.89 ± 0.58 ^c^	5.88 ± 0.49
Bifidobacteria	5.94 ± 0.17	5.21 ± 0.54	6.97 ± 0.08	4.75 ± 0.22	6.74 ± 0.03	5.84 ± 0.66	7.02 ± 0.74	6.29 ± 0.90
Lactobacilli	4.58 ± 0.42	5.15 ± 0.65	6.85 ± 0.11 ^a^	5.46 ± 0.38	4.09 ± 0.04 ^b^	5.45 ± 0.15	6.70 ± 0.91 ^c^	5.58 ± 0.32

Data are expressed as mean ± SD (n = 6 per group). ^a^ *p* < 0.001 compared to CD, ^b^ *p* < 0.001 compared to CCD, and ^c^ *p* < 0.001 compared to DCD. CD: animals that followed the control diet; CCD: animals that followed the Corinthian currant diet; DCD: diabetic animals that followed the control diet; DCCD: diabetic animals that followed the Corinthian currant diet; TAC: total aerobic counts.

**Table 5:** Taxonomic-binning at the phylum level with normalized relative abundances (%) after four weeks of Corinthian currant supplementation in control and STZ-induced diabetic rat feces, as determined by 16S rRNA NGS (n = 2 per group).

Phylum	Relative Abundance (%)							
	CD		CCD		DCD		DCCD	
	Baseline	End	Baseline	End	Baseline	End	Baseline	End
Firmicutes	71.52 ± 3.59	69.69 ± 6.19	68.08 ± 2.61	72.60 ± 6.14	72.86 ± 1.71	75.99 ± 6.13	73.20 ± 6.80	71.14 ± 3.53
Bacteroidetes	22.63 ± 4.31	27.58 ± 2.69	20.89 ± 2.21	19.09 ± 2.33	25.16 ± 0.72	21.56 ± 4.38	20.07 ± 1.82	24.54 ± 8.22
Actinobacteria	0.03 ± 0.01	0.05 ± 0.02	0.07 ± 0.04	0.10 ± 0.06	1.82 ± 2.53	2.29 ± 1.86	0.13 ± 0.03	1.90 ± 2.10
Verrucomicrobia	0.13 ± 0.11	0.14 ± 0.06	9.69 ± 0.23	5.69 ± 4.96	0.11 ± 0.04	0.08 ± 0.01	6.35 ± 5.85	2.47 ± 2.29
Tenericutes	0.09 ± 0.01	0.18 ± 0.13	1.27 ± 0.12	2.52 ± 0.57 ^#^	0.05 ± 0.04	0.09 ± 0.12	0.25 ± 0.19	0.13 ± 0.11

Values are expressed as mean ± SD. ^#^ *p* < 0.01 compared to baseline values. CD: animals that followed the control diet; CCD: animals that followed the Corinthian currant diet; DCD: diabetic animals that followed the control diet; DCCD: diabetic animals that followed the Corinthian currant diet.

**Table 6:** Effect of diets on lactic acid and SCFA concentrations (μmol/g) in the feces of control and STZ-induced diabetic rats.

	CD		CCD		DCD		DCCD	
	Baseline	End	Baseline	End	Baseline	End	Baseline	End
Lactic acid	1.70 ±0.52	1.16 ± 0.02	1.79 ± 0.52	1.66 ± 0.53	1.94 ± 0.57	1.75 ± 0.52	2.01 ± 0.53	1.52 ± 0.22
Acetic acid	19.55 ± 5.56	24.25 ± 5.45	17.65 ± 5.10	30.48 ± 4.49 ^#^	34.81 ± 7.03 ^a,b^	24.49 ± 9.09	34.78 ± 8.10 ^a,b^	56.08 ± 9.86 ^a,b,#^
Propionic acid	1.93 ± 0.92	1.67 ± 0.46	2.00 ± 0.99	1.80 ± 0.19	1.89 ± 0.43	2.57 ± 0.86	1.78 ± 0.30	2.41 ± 0.75
Isobutyric acid	0.09 ± 0.05	0.29 ± 0.05 ^#^	0.10 ± 0.04	0.31 ± 0.14 ^#^	0.07 ± 0.02	0.20 ± 0.10 ^#^	0.07 ± 0.03	0.32± 0.06 ^#^
Butyric acid	1.41 ± 0.86	1.33 ± 0.68	1.28 ± 0.79	1.94 ± 0.39	1.37 ± 0.55	1.53 ± 0.71	1.43 ± 0.57	1.48 ± 0.91
Isovaleric acid	0.06 ± 0.01	0.12 ± 0.04	0.06 ± 0.01	0.11 ± 0.02	0.04 ± 0.02	0.10 ± 0.06	0.04 ± 0.02	0.10 ± 0.06
Valeric acid	0.11 ± 0.02	0.15 ± 0.03	0.10 ± 0.02	0.16 ± 0.04	0.10 ± 0.02	0.17 ± 0.07	0.10 ± 0.02	0.19 ± 0.09

Values are expressed as mean ± SD (n = 6 per group). ^a^ *p* < 0.001 compared to CD, ^b^ *p* < 0.001 compared to CCD, and ^#^ *p* < 0.01 compared to baseline values. CD: animals that followed the control diet; CCD: animals that followed the Corinthian currant diet; DCD: diabetic animals that followed the control diet; DCCD: diabetic animals that followed the Corinthian currant diet.

## Data Availability

The data presented in this study are available in the main article and the Supplementary Material.

## Supplementary Materials

The following supporting information can be downloaded at: https://www.mdpi.com/article/10.3390/metabo13030415/s1. Table S1: Polar phenolic content of Corinthian currant and rat chow title; Table S2: Two-way ANOVA parameters for main effects and associated interactions for the detected levels of polar phenolics in the serum of rats; Table S3: Nutrients’ composition and energy content of rat chow and Corinthian currants provided to the animals.
