# Supplementary material for: Corinthian Currants Supplementation Restores Serum Polar Phenolic Compounds, Reduces IL-1beta, and Exerts Beneficial Effects on Gut Microbiota in the Streptozotocin-Induced Type-1 Diabetic Rat
Source: Metabolites. 2023 Mar 11;13(3):415. doi: 10.3390/metabo13030415 (PMC10051135; doi:10.3390/metabo13030415)
Supplement: Supplementary file 1 [file metabolites-13-00415-s001.zip › metabolites-2204615-supplementary.pdf]

Supplementary Material

Table S1. Polar phenolic content of Corinthian currant and rat chow.

**Polar Phenol (μg/ 100g)**	**Rat Chow**	**Corinthian currant**
	**Flavonols**	
Isorhamnetin	0.8 ± 0.2	27.8 ± 1.5
Kaempferol	12.7 ± 2.1	32.4 ± 4.8
Quercetin	4.4 ± 1.2	167.4 ± 19.7
	**Flavones**	
Apigenin	31.8 ± 3.5	tr
Chrysin	n.d.	0.2 ± 0.03
Luteolin	29.7 ± 8.1	1.10 ± 0.1
	**Flavanones**	
Hesperetin	1.28 ± 0.3	0.51 ± 0.11
Naringenin	2.6 ± 0.9	1.96 ± 0.33
	**Isoflavones**	
Daidzein	137.1 ± 13.7	tr
Formononetin	8.2 ± 1.1	tr
Genistein	299.6 ± 47.2	0.11 ± 0.1
	**Benzoic acid derivatives**	
Vanillic acid	47.1 ± 9.4	241.5 ± 30.2
Syringic acid	40.4 ± 5.3	102.6 ± 14.9
	**Cinnamic acid derivatives**	
trans-Cinnamic acid	n.d.	6.02 ± 0.3

Table S2. Two-way ANOVA parameters for main effects and associated interactions for the detected levels of polar phenolics in the serum of rats.

		Flavonols					
	df	Isorhamnetin		Kaempferol		Quercetin	
		F	P	F	P	F	P
**Corrected model**	3	95.69	<0.001	49.34	<0.001	42.06	<0.001
**Intercept**	1	139.55	<0.001	335.96	<0.001	58.17	<0.001
**Treatment**	1	102.53	<0.001	9.63	0.005	44.85	<0.001
**Food**	1	108.41	<0.001	131.64	<0.001	46.57	<0.001
**Treatment x Food**	1	76.12	<0.001	6.75	0.016	34.75	<0.001

		Flavones					
	df	Apigenin		Chrysin		Luteolin	
		F	P	F	P	F	P
**Corrected model**	3	102.68	<0.001	113.80	<0.001	27.72	<0.001
**Intercept**	1	460.93	<0.001	188.24	<0.001	84.45	<0.001
**Treatment**	1	34.37	<0.001	76.58	<0.001	5.33	0.030
**Food**	1	272.43	<0.001	188.24	<0.001	74.18	<0.001
**Treatment x Food**	1	1.26	0.273	76.58	<0.001	3.65	0.068

		Flavanones			
	df	Hesperetin		Naringenin	
		F	P	F	P
**Corrected model**	3	50.10	<0.001	27.06	<0.001
**Intercept**	1	297.40	<0.001	244.81	<0.001
**Treatment**	1	3.78	0.064	0.83	0.370
**Food**	1	145.89	<0.001	78.78	<0.001
**Treatment x Food**	1	0.64	0.431	1.57	0.223

		Isoflavones					
	df	Daidzein		Formononetin		Genistein	
		F	P	F	P	F	P
**Corrected model**	3	47.80	<0.001	42.53	<0.001	35.24	<0.001
**Intercept**	1	266.22	<0.001	247.94	<0.001	211.47	<0.001
**Treatment**	1	5.30	0.030	20.29	<0.001	4.07	0.055
**Food**	1	126.72	<0.001	107.28	<0.001	98.02	<0.001
**Treatment x Food**	1	11.36	0.003	0.03	0.854	3.63	0.069

		Benzoic acid derivatives				Cinnamic acid derivatives	
	df	Vanillic acid		Syringic acid		trans-cinnamic acid	
		F	P	F	P	F	P
**Corrected model**	3	192.66	<0.001	34.42	<0.001	8.17	0.001
**Intercept**	1	1627.82	<0.001	211.46	<0.001	200.75	<0.001
**Treatment**	1	183.13	<0.001	24.50	<0.001	1.74	0.199
**Food**	1	84.66	<0.001	3.27	0.083	19.01	<0.001
**Treatment x Food**	1	310.20	<0.001	75.49	<0.001	3.76	0.064

Table S3. Nutrients' composition and energy content of rat chow and Corinthian currants provided to the animals.

Nutrients	Rat Chow	Corinthian currants
Macronutrients (g/ 100g)		
Proteins	18.5	2.5
Fat	3.0	< 0.4
Carbohydrates	46.3	77.5
Dietary fiber	6.0	6.7
Energy (Kcal)	387.6	294
Minerals (mg/ 100g)		
Ca	. 101% U.M.	10
P	0.57 % U.M.	181
Na	0.3 % U.M.	-
Cl	0.26 % U.M.	-
K	0.61 % U.M.	710
Mg	0.17 % U.M.	30
Fe	33.2	4
Cu	1.98	-
Mn	7.37	-
Zn	8.71	0.6
Co	0.056	-
I	0.063	-
Se	0.013	-
Vitamins (mg/100g)		
A	15400 (IU/kg)	-
D3	1340 (IU/kg)	-
E	6.3	-
B1 (thiamine)	1.66	0.14
B2 (riboflavin)	.813	0.11
B6 (pyridoxine)	.883	0.19
B12 (cyanocobalamin)	0.003	-
K (menadione)	0.311	-
Folic acid	.229	6.6
Nicotinic acid	8.51	1.01
Pantothenic acid	2.01	n.d.
Choline	177.1	-
Biotin	.035	n.d.

U.M.: measurement unit, n.d.: not detected.
